# Supplementary material for: SARS-CoV-2 viral load as a predictor for disease severity in outpatients and hospitalised patients with COVID-19: A prospective cohort study
Source: PLoS One. 2021 Oct 12;16(10):e0258421. doi: 10.1371/journal.pone.0258421 (PMC8509867; doi:10.1371/journal.pone.0258421)
Supplement: S1 Appendix — (DOCX) [file pone.0258421.s001.docx]

**S1 Appendix 1** from article “SARS-CoV-2 viral load as a predictor for disease severity in outpatients and hospitalised patients with COVID-19: a prospective cohort study” by Fredrikke Christie Knudtzen, Thøger Gorm Jensen, Susan Olaf Lindvig, Line Dahlerup Rasmussen, Lone Wulff Madsen, Silje Vermedal Hoegh, Malene Bek-Thomsen, Christian B. Laursen, Stig Lønberg Nielsen and Isik Somuncu Johansen.

**Questionnaire**

**The COVID-19 Outpatient Cohort – Region of Southern Denmark**

The questionnaire was sent out electronically to the patients’ electronic mailboxes on May 15^th^ 2020. If they did not respond they received an electronic reminder on May 22th and a text message on May 29^th^. The opportunity to respond shut down on June 1^st^ 2020.

The questionnaire consisted of the following questions:

Full name

SSN [Danish personal identification number]

Date of birth

Sex

Age at symptom debut (automatically calculated)

BMI (automatically calculated)

Baseline information

Height in cm

Weight in kg

Smoking status

- Never smoked
- Previously smoked
- Currently smoking
- I do not want to reply

Is your current use of alcohol below or above the recommended limit of the Danish Health authority? The Danish Health Authority recommends less than 7 units per week for women and 14 units per week for men.

- Below
- Above
- I do not want to reply

What is your level of education?

- Primary and secondary lower school
- High school/vocational upper secondary education
- Short-cycle higher education
- Medium-cycle higher education
- Long-cycle higher education

Do you work in the health care system?

- No
- Yes
- I do not want to reply

Do you have any known diseases?

Do you have or have you had any of the following cardiovascular diseases?

- No
- Myocardial infarction (MI)
- Heart arrhythmias
- Heart insufficiency
- Heart valve disease
- Other cardiovascular disease (possible to leave a comment)
- I do not want to reply

Do you have or have you had any of the following pulmonary diseases?

- No
- COPD
- Asthma
- Other lung disease (possible to leave a comment)
- I do not want to reply

Do you have or have you had any of the following rheumatic diseases?

- Rheumatoid arthritis
- Other rheumatic disease (possible to leave a comment)
- I do not want to reply

Do you have hypertension?

- No
- Yes
- I do not want to reply

Do you have hypercholesterolemia?

- No
- Yes
- I do not want to reply

Do you have diabetes?

- No
- Yes
- I do not want to reply

Do you have a kidney disease?

- No
- Yes
- I do not want to reply

Do you have or have you had any of the following gastrointestinal diseases?

- No
- Ulcer
- Crohn’s disease/Ulcerative colitis
- Other gastrointestinal disease (possible to leave a comment)

Do you have or have you had cancer?

- No
- Yes (possible to leave a comment)
- I do not want to reply

Do have any other diseases?

- No
- Yes (possible to leave a comment)
- I do not want to reply

Are you currently taking any medication?

- No
- Yes (pop-up box asking about name of medication)
- I do not want to reply

Exposure

Have you visited a foreign country with 14 days of symptom debut?

- No
- Yes (pop-up box with the possibility to choose among China, Italy, Iran, South Korea, Austria, France, Spain or other (and to leave a comment on which region) )
- I do not want to reply

Have you, within 14 days of symptom debut, had close physical contact with one or more people who have visited a foreign country? (Close contact is defined as distance of less than 1 meter in 15 minutes or less)

- No
- Yes
- I do not want to reply

Have you, within 14 days of symptom debut, had close physical contact with one or more people who are suspected or tested positive for coronavirus? (Close contact is defined as distance of less than 1 meter in 15 minutes or less)

- No
- Yes
- I do not want to reply

If yes to the above, who have you been in contact with? (Possible to choose more than one option)

- One or more people from my household
- Co-worker
- Social gathering
- Other (possible to leave a comment)

Contact at what date?

- Enter date

What sex do the person/persons who you think infected you have? (Possible to choose more than one option)

- Man
- Woman
- I do not know

What age do the person/persons who you think infected you have?

- Child aged 0-5 years
- Child aged 6-12
- Teenager aged 13-19
- Adult aged 20-40
- Adult over 40 years old
- I do not know

At what date did your symptoms start?

- Enter date

What symptoms did you have?

- Fever
- Cough
- Dyspnea
- Headache
- Dizziness
- Sore throat
- Stuffy nose
- Myalgia
- Nausea/vomiting
- Stomach pain
- Diarrhea
- Change/loss of smell
- Change/loss of taste
- Fatigue

Except for fatigue, dyspnea, and changes/loss in taste and smell, when did your symptoms stop?

- Enter date

Was it possible to self-isolate during the course of the illness?

- No
- Yes
- I do not want to reply

How many days were you on sick-leave from work/education?

- Enter number of days

Are you back to work/education in the same capacity and same number of work hours as before?

- No
- Yes
- I do not want to reply

In your opinion, are you able to concentrate as well as before the coronavirus?

- No
- Yes
- I do not know

In your opinion, are you able to remember as well as before the coronavirus?

- No
- Yes
- I do not know

In your opinion, do you feel more fatigued than before the coronavirus?

- No
- Yes
- I do not know

Have you been worried that you would suddenly become seriously ill? (Grade from 0-10. 0 not worried, 10 being very worried)

Have you been worried, that someone close to you would suddenly become seriously ill? (Grade from 0-10. 0 being not worried, 10 being very worried)

Compared to previous upper respiratory infections, how have you experienced you degree of illness? (Grade from 0-10. 0 less ill then usually, 10 more ill then usually)

Have you been vaccinated for seasonal influenza within the past 10 years?

- No
- Yes
- I do not want to reply

Do you plan to get vaccinated for seasonal influenza this year?

- No
- Yes
- I do not want to reply
