# Supplementary material for: SARS-CoV-2 viral load as a predictor for disease severity in outpatients and hospitalised patients with COVID-19: A prospective cohort study
Source: PLoS One. 2021 Oct 12;16(10):e0258421. doi: 10.1371/journal.pone.0258421 (PMC8509867; doi:10.1371/journal.pone.0258421)
Supplement: S1 Table — (DOCX) [file pone.0258421.s002.docx]

**S1 Table.** Logistic regression model displaying univariate and multivariate estimates of risk factors associated with hospital admission in patients with SARS-Co-V-2.

|  | Unadjusted | | Adjusted | |
| --- | --- | --- | --- | --- |
|  | Odds ratio (95% CI) | p-value | Odds ratio (95% CI) | p-value |
| C_q_-value* | 1.11 (1.04-1.19) | 0.002 | 1.08 (0.94-1.24) | 0.273 |
| Sex | 1.28 (0.68-2.41) | 0.444 |  |  |
| Age* | 1.11 (1.07-1.15) | <0.001 | 1.09 (1.04-1.15) | 0.001 |
| BMI* | 1.12 (1.05-1.20) | 0.001 | 1.21 (1.06-1.38) | 0.004 |
| Comorbidities  Cardiovascular disease  Hypertension  Diabetes mellitus type I+II  Malignancies | 4.90 (2.49-9.65)  3.52 (1.74-7.16)  15.83 (2.02-124.01)  3.29 (1.13-9.61) | <0.001  <0.001  0.009  0.029 | 12.8 (1.26-130.04)  0.09 (0.01-0.97)  23.01 (0.75-708.62)  1.97 (0.36-10.71) | 0.031  0.047  0.073  0.432 |
| Time from symptom onset to testing | 1.92 (1.57-2.36) | <0.001 | 2.05 (1.53-2.74) | <0.001 |

CI=confidence interval; C_q_-value=SARS-CoV-2 polymerase chain reaction cycle quantification value; BMI=body mass index

*C_q_-values, age and BMI have been analysed as continuous variables
